# Clinicopathological characteristics and survival outcomes in patients with angiosarcoma of breast

**DOI:** 10.1002/cam4.6042

**Published:** 2023-05-04

**Authors:** Junfeng Li, Yunhai Li, Yuanyuan Wang, Zhao Li, Huan Zhang, Yidan Gao, Jinxiang Tan

**Affiliations:** ^1^ Department of Breast and Thyroid Surgery The First Affiliated Hospital of Chongqing Medical University Chongqing China

**Keywords:** angiosarcoma, breast, risk factor, survival, treatment

## Abstract

**Background:**

Angiosarcoma of the breast is a rare malignancy. There are little data evaluating the survival and estimating the prognostic factors. The best surgical management and the role of systemic adjuvant therapy remain ill‐defined. This study aimed to investigate the clinicopathological features, survival, and prognostic factors of breast angiosarcoma.

**Methods:**

The data on patients diagnosed with breast angiosarcoma were extracted from the Surveillance, Epidemiology, and End Results database (1975–2016). Univariate and multivariate Cox regression analyses were used to estimate the influential prognostic factors. The overall survival (OS) and disease‐specific survival (DSS) of patients with breast angiosarcoma were evaluated.

**Results:**

This study included 656 patients diagnosed with breast angiosarcoma between 1975 and 2016. The 5‐year OS rate of all patients was 44.9% (95% CI 40.8–49.0). In both OS and DSS, Kaplan–Meier survival analyses revealed significant differences for both OS and DSS according to age, year at diagnosis, laterality, grade, and stage (all log‐rank *p* < 0.05). Multivariate analysis suggested that lesions of the right breast, poor differentiation, and advanced stage were independent risk factors for OS or DSS (all *p* < 0.05). Older age was a risk factor in OS, but was protective in DSS. In primary breast angiosarcoma, age, laterality, grade, and stage were independent prognostic factors in OS and DSS (all *p* < 0.05). Mastectomy was also a risk factor in DSS (*p* = 0.034). The proportion of patients with grade III and regional disease was larger in the mastectomy group.

**Conclusion:**

Angiosarcoma of the breast had a poor prognosis. In our study, age, laterality, histologic grade, and stage were identified as significant prognostic factors. Why patients with angiosarcoma of the right breast had a worse prognosis remains equivocal. Mastectomy was adopted more often by surgeons in this cohort study for patients with advanced primary breast angiosarcoma.

## 1 INTRODUCTION

Angiosarcoma is a malignant tumor that originates from endothelial cells. These tumors can occur in different parts of the body.^1^ Angiosarcoma can be divided into de novo (primary) and therapy‐related (secondary) diseases. Current commonly recognized causes of secondary angiosarcoma include radiation exposure and chronic lymphedema (also termed Stewart–Treves syndrome).^1^ Currently, the comprehensive treatment of breast cancer, radiotherapy, and axillary lymph node dissection occupy an important position. Accordingly, many reports take angiosarcoma of the breast region as a representative to analyze secondary angiosarcoma.^2^

Angiosarcoma of the breast is an extremely rare and heterogenous soft‐tissue tumor, which accounts for <1% of total breast malignancies.^3^ Primary and secondary breast angiosarcoma differ in age of onset and some clinical circumstances. In general, compared with secondary breast angiosarcoma, primary breast angiosarcoma is considered to occur in younger women with a better prognosis, larger tumor size, and no previous related history.^4^, ^5^ Nevertheless, modern medical institutions adopt the same treatment management for both primary and secondary angiosarcoma, emphasizing complete surgical excision with optimal margin.^6^ The status of chemotherapy and radiotherapy in management strategies remains controversial.^7^

The relevant literature that is available is mainly case reports, meta‐analyses, and single‐center retrospective studies with a limited number of cases. Thus, in the present study, we retrospectively analyzed the clinical data of larger‐scale breast angiosarcoma patients obtained from the Surveillance, Epidemiology, and End Results (SEER) database to study the survival outcomes and prognostic factors. This study strategy reflected the rarity of the disease, but the varied survival outcomes between primary and secondary tumors.^8^ Our use of the large SEER database of primary breast angiosarcoma patients was done to more definitively explore the clinical features and survival outcomes of angiosarcoma in terms of breast region from a broader perspective.

## 2 MATERIALS AND METHODS

### 2.1 Data source

The data were from the SEER database of the National Cancer Institute. SEER*Stat, version 8.3.9 is highly accurate with high positive predictive value,^9^ and so was used to generate the case listing. The SEER database consists of 18 population‐based cancer registries and captures approximately 28% of the United States population.^10^ All included data represent the latest follow‐up (March 15, 2021) available in the SEER database.

### 2.2 Patients

All patients were diagnosed from 1975 to 2016. Patients' clinicopathological features and survival data were extracted by International Classification of Disease for Oncology (ICD‐O) site recode 3 (breast) and histologic subtype ICD‐O‐3 (angiosarcoma 9120/3). The variables in the case listing included patient identification, age at diagnosis, sex, race recode, year of diagnosis, laterality, grade, stage, surgery, chemotherapy recode, radiotherapy recode, survival months, vital status, and cause of death to site recode. Considering the importance of predicting survival,^8^ tumor size was also initially included in this case listing. However, this variable was excluded because of the huge amount of missing data (640/672). A total of 672 SEER registry patients diagnosed with breast angiosarcoma from 1975 to 2016 were identified. Patients with duplicate patent identification and unknown survival months were excluded. Finally, 656 patients were eligible for this study (Figure S1). Of these patients, 264 with primary breast angiosarcoma were selected from this data through SEER sequence number. Only those patients who were recorded as one primary only and the first of two or more primaries were considered to have primary breast angiosarcoma. It is important to note some variables in this study. According to the dictionary of SEER*Stat Variables (https://seer.cancer.gov/data‐software/documentation/seerstat/nov2018), the grade system was used to classify the tumor into well‐differentiated (grade I), moderately differentiated (grade II), poorly differentiated (grade III), undifferentiated (grade IV), or unknown based on ICD‐O‐3. Also in the dictionary, the SEER historic stage A was used to categorize the cases as local, regional, distant, or unknown, which is derived from Collaborate Stage and Extent of Disease.

### 2.3 Statistical analyses

Descriptive statistics were used to examine the baseline characteristics of the 656 patients with breast angiosarcoma. For the laterality group, the *χ* ^2^ test was used to compare the differences in patient characteristics between the left and right sites. The *χ* ^2^ test was also used to explore the differences in the constituent ratio of different literalities and different surgical methods in patients with primary breast angiosarcoma. Overall survival (OS) was defined as the time from diagnosis of breast angiosarcoma to death due to any cause or the follow‐up cutoff. Disease‐specific survival (DSS) was calculated from the diagnosis of breast angiosarcoma to death of this disease. Kaplan–Meier survival curve and log‐rank tests were conducted to compare OS and DSS among different groups. In the part of primary breast angiosarcoma, Kaplan–Meier survival curve, and log‐rank tests were used to compare the survival of patients with different surgical methods. For both OS and DSS, univariate and multivariate Cox proportional hazard models were performed to assess the influences of age at diagnosis, race, year of diagnosis, laterality, grade, stage, surgery, chemotherapy, and radiotherapy on hazard ratios (HRs). The 95% confidence intervals (CIs) were computed for 5‐year OS, 5‐year DSS, and HRs. All variables in our study were assigned with Microsoft Excel 2016 MSO (16.0.13901.20148) 32bit. All statistical analyses were performed using IBM SPSS Statistics Version 26 software. All figures were drawn by GraphPad Prism 8.0.2 software. A *p*‐value ≤0.05 was considered statistically significant. All tests in our study were 2‐tailed.

## 3 RESULTS

### 3.1 Patient characteristics

In the search of the SEER database, 656 patients met the inclusion criteria. The demographic and clinical characteristics are summarized in Table S1. The mean age at diagnosis was 68 years (range 15–96 years). Most of the patients were white (86.43%). Only three patients (0.46%) were men in this cohort. The lesions in 337 (51.37%) patients were located in the left breast and 317 (48.32%) were in the right breast. Two patients (0.30%) had lesions in both breasts, but only one side was the primary lesion. More than half of patients (52.59%) presented with localized disease. The majority of the patients (73.17%) underwent mastectomy. Most did not receive chemotherapy or radiotherapy (75.30% and 80.95%, respectively). At the end of the follow‐up, 257 patients were still alive and 107 patients were dead due to the breast angiosarcoma. More detailed characteristics of patients are provided in Table S1.

### 3.2 Overall survival

**FIGURE 1:**
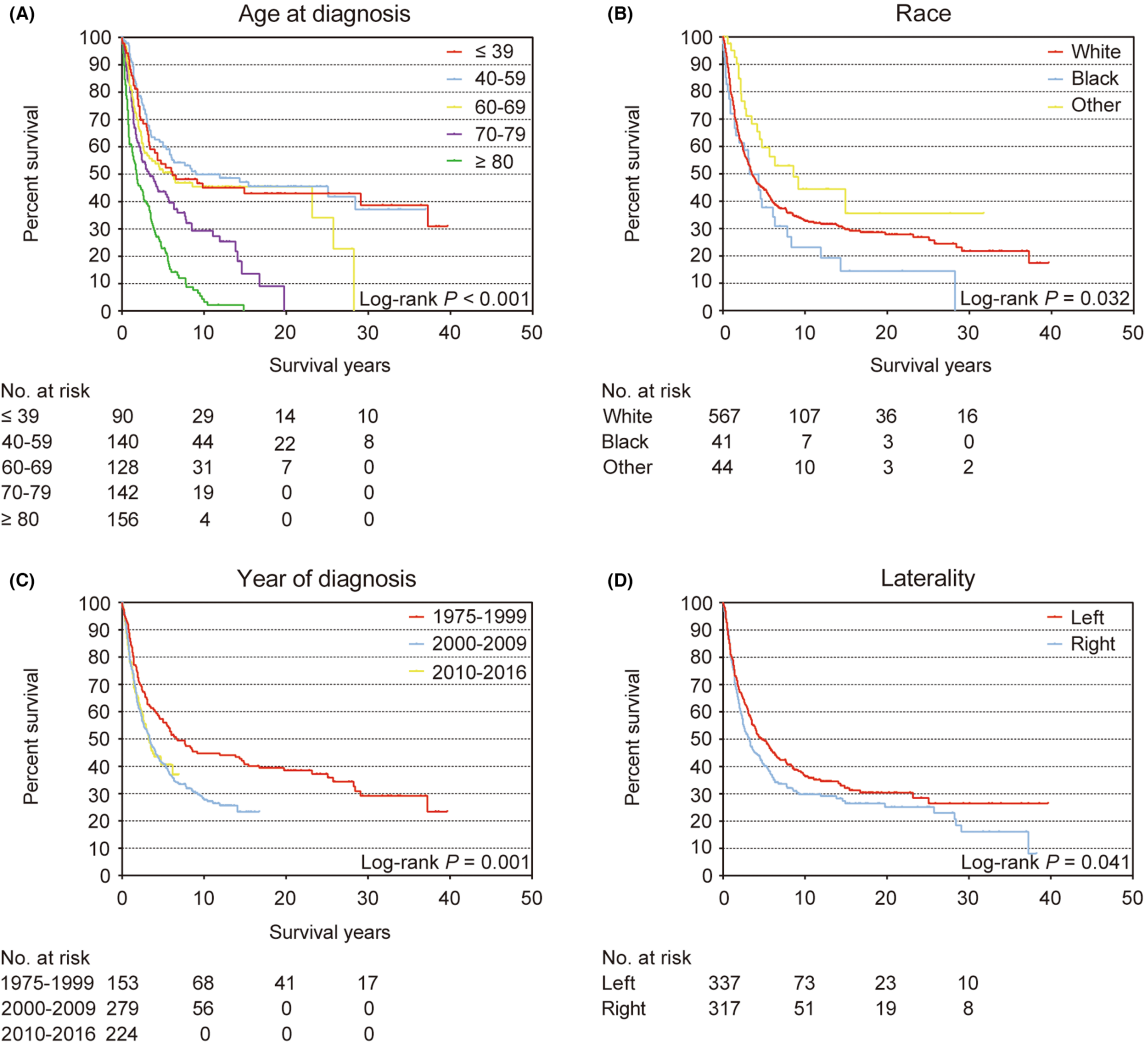
Kaplan–Meier survival curves for clinical features in overall survival. (A) Age; statistically significant differences are evident among ≤39, 40–59, 60–69, 70–79, and ≥80 age groups (*p* < 0.001). (B) Race; statistically significant differences are evident among white, black, and other (*p* = 0.032). (C) Year of diagnosis; statistically significant differences are evident among patients diagnosed in 1975–1999, 2000–2009, and 2010–2016 (*p* = 0.001). (D) Laterality; statistically significant differences are evident between patients with left and right lesions (*p* = 0.031).

**FIGURE 2:**
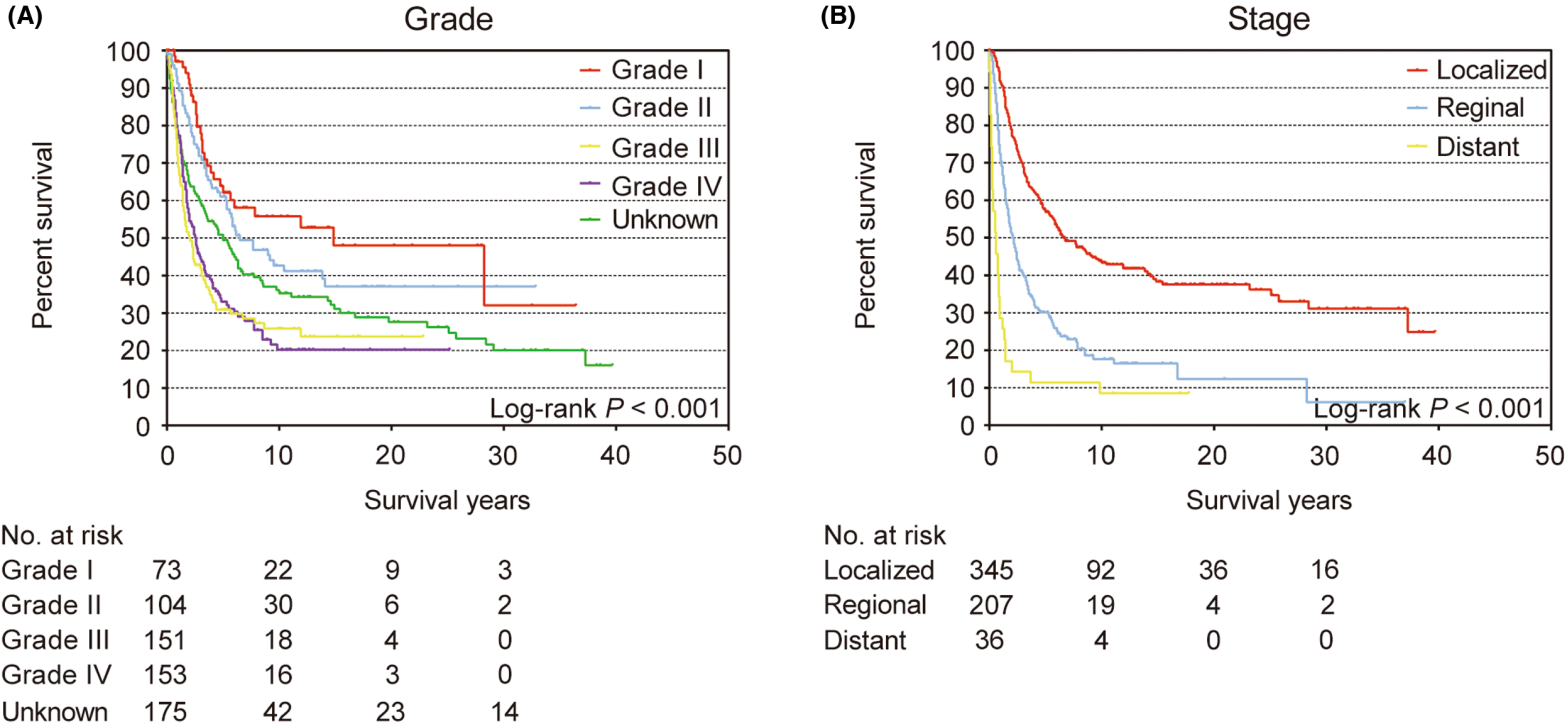
Kaplan–Meier survival curves for oncology characteristics in overall survival. (A) Grade; statistically significant differences are evident among group grade I, grade II, grade III, grade IV, and unknown (*p* < 0.001). (B) Stage; statistically significant differences are evident among localized, regional, and distant groups of patients (*p* < 0.001).

The 5‐year OS rate of all patients was 44.9% (95% CI 40.8–49.0) (Figure S2). The Kaplan–Meier survival analysis revealed statistically significant differences in age, race, year at diagnosis (Figure 1), grade, stage (Figure 2), and surgery (Figure S3) (all log‐rank *p* < 0.05). Interestingly, there was also a statistically significant difference in OS between the left and right breasts (log‐rank *p* = 0.041) (Figure 1D). Compared with patients whose lesion was in left breast (49.4%, 95% CI 39.2–55.1), patients with right breast lesions had a worse 5‐year OS (40.1%, 95% CI 34.2–46.0). The unadjusted HR for patients with right breast angiosarcoma was 1.26 (95% CI 1.01–1.49; *p* = 0.043) (Table 1). Chemotherapy and radiotherapy were not associated with improved survival. No statistically significant difference was found in Kaplan–Meier survival analysis of chemotherapy (log‐rank *p* = 0.86) or radiotherapy group (log‐rank *p* = 0.86) (Figure S3).

**TABLE 1:** Five‐year overall survival (OS) and hazard ratios (HRs) for OS in breast angiosarcoma.

Variable	5‐year OS (95% CI)	Univariate analysis			Multivariate analysis		
		HR	95% CI	*p*‐value	HR	95% CI	*p*‐value
Age of diagnosis (years)							
≤39	53.7% (42.7–64.7)	1.00	Reference		1.00	Reference	
40–59	60.0% (51.4–68.6)	0.92	0.63–1.34	0.66	0.91	0.61–1.35	0.63
60–69	50.6% (41.4–59.8)	1.24	0.84–1.82	0.28	1.10	0.72–1.66	0.666
70–79	43.6% (34.8–52.4)	1.85	1.28–2.66	0.001	1.78	1.19–2.68	0.006
≥80	22.8% (15.5–30.1)	3.29	2.32–4.67	<0.001	2.92	1.95–4.36	<0.001
Race							
White	44.1% (39.8–48.4)	1.00	Reference		1.00	Reference	
Black	37.7% (21.6–53.8)	1.29	0.89–1.88	0.18	1.63	1.10–2.42	0.015
Other	59.6% (43.7–75.5)	0.62	0.39–0.97	0.02	0.78	0.49–1.23	0.28
Diagnosis (years)							
1975–1999	56.0% (48.2–63.8)	1.00	Reference		1.00	Reference	
2000–2009	40.8% (34.9–46.7)	1.57	1.22–2.03	<0.001	1.01	0.75–1.36	0.93
2010–2016	40.6% (32.0–49.2)	1.53	1.14–2.06	0.005	0.99	0.70–1.38	0.93
Laterality							
Left	49.4% (39.2–55.1)	1.00	Reference		1.00	Reference	
Right	40.1% (34.2–46.0)	1.26	1.01–1.49	0.043	1.34	1.09–1.64	0.005
Grade							
I	62.1% (49.8–74.4)	1.00	Reference		1.00	Reference	
II	61.1% (51.3–70.9)	1.35	0.86–2.11	0.20	1.55	0.98–2.47	0.064
III	30.9% (22.5–39.3)	2.74	1.80–4.16	<0.001	2.17	1.39–3.38	0.001
IV	33.1% (25.1–41.1)	2.60	1.72–3.93	<0.001	1.98	1.28–3.07	0.002
Unknown	49.4% (41.6–57.2)	1.87	1.25–2.82	0.03	1.71	1.11–2.63	0.015
Stage							
Localized	56.9% (51.4–62.4)	1.00	Reference		1.00	Reference	
Regional	30.1% (23.6–36.6)	2.25	1.81–2.79	<0.001	1.60	1.27–2.01	<0.001
Distant	11.1% (0.8–22.0)	5.22	3.57–7.62	<0.001	4.75	3.07–7.35	<0.001
Unknown	52.3% (35.2–69.4)	1.31	0.86–2.01	0.21	1.36	0.84–2.20	0.21
Surgery							
No	30.5% (15.4–45.6)	1.00	Reference		1.00	Reference	
Conserving surgery	59.9% (49.1–70.7)	0.46	0.29–0.73	0.001	0.66	0.40–1.12	0.12
Mastectomy	42.7% (38.0–47.4)	0.67	0.46–0.98	0.04	0.84	0.54–1.30	0.42
Surgery, NOS	53.3% (36.6–70.0)	0.49	0.28–0.85	0.011	0.80	0.43–1.50	0.48
Chemotherapy							
No/unknown	46.6% (41.9–51.3)	1.00	Reference		1.00	Reference	
Yes	39.4% (31.0–47.8)	1.02	0.81–1.30	0.86	1.00	0.77–1.31	0.98
Radiotherapy							
No/unknown	45.8% (41.3–50.3)	1.00	Reference		1.00	Reference	
Yes	41.2% (32.0–50.4)	1.02	0.80–1.31	0.87	1.16	0.88–1.52	0.30

Abbreviations: OS, overall survival; NOS, not otherwise specified.

In multivariate analysis (Table 1), regarding the impact of age on OS, compared to patients ≤39 years of age, 5‐year OS was worse for patients diagnosed at 70–79 years of age (43.6% vs. 53.7%; HR 1.78, 95% CI 1.19–2.68; *p* = 0.006) and ≥80 years of age (22.8% vs. 53.7%; HR 2.92, 95% CI 1.95–4.36; *p* < 0.001). Black race was also a significant independent risk factor of OS in patients with breast angiosarcoma (HR 1.63, 95% CI 1.10–2.42; *p* = 0.015). Consistent with the Kaplan–Meier survival and univariate analyses, the multivariate analysis revealed significant differences between patients with different laterality. The HR for the right lesion group compared with right group was 1.34 (95% CI 1.09–1.64; *p* = 0.005). In addition, taking the well‐differentiated group and localized group as the classification reference, grade, and stage were independent risk factors affecting prognosis. HR was 2.17 (95% CI 1.39–3.38; *p* = 0.001) for patients with grade III disease and 1.98 (95% CI 1.28–3.07; *p* = 0.002) for those with grade IV disease. The prognosis was dismal for patients with regional disease (HR 1.60, 95% CI 1.27–2.01; *p* < 0.001), and distant disease had performed dismal prognosis (HR 4.75, 95% CI 3.07–7.35; *p* < 0.001).

### 3.3 Disease‐specific survival

**FIGURE 3:**
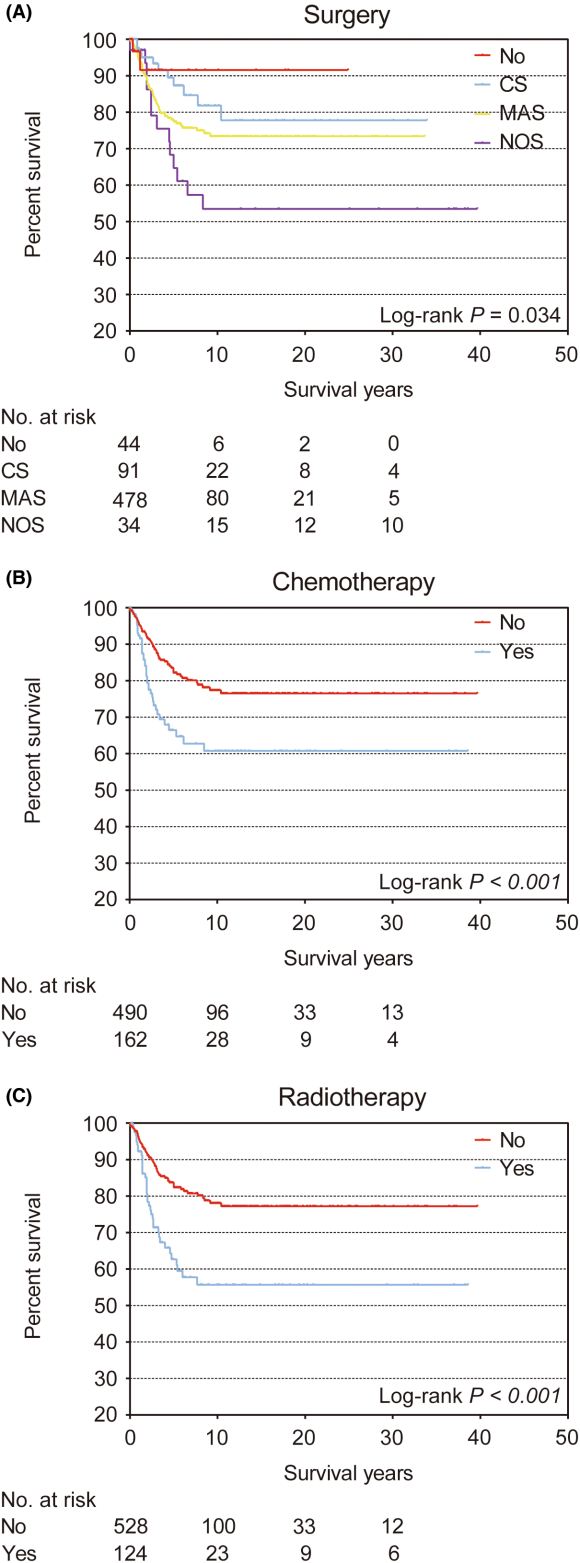
Kaplan–Meier survival curves for treatment in disease‐specific survival. (A) Surgery; statistically significant differences are evident among no surgery (No), conserving surgery (CS), mastectomy (MAS), and not otherwise specified (NOS) groups (*p* = 0.034). (B) Chemotherapy; statistically significant differences are evident between patients who received chemotherapy and those who did not (*p* < 0.001). (C) Radiotherapy; statistically significant differences are evident between patients who received radiotherapy and those who did not (*p* < 0.001).

The 5‐year DSS of the entire cohort was 78.3% (95% CI 74.2–82.4) (Figure S2). In the Kaplan–Meier survival curves, age, year at diagnosis, laterality (Figure S4), grade, stage (Figure S5), surgery, chemotherapy, and radiotherapy (Figure 3) presented with statistically significant differences (all log‐rank *p* < 0.05). Race did not show statistical differences, which was inconsistent with the OS data (log‐rank *p* = 0.802). The 5‐year DSS of patients with higher age was more optimistic. Compared with patients ≤39 years (5‐year DSS: 57.2%, 95% CI 45.8–68.6), the 5‐year DSS was 79.9% (95% CI 70.9–88.9) in patients 60–69 years of age, 94.8% (95% CI 90.7–98.9) in patients 70–79 years, and 90.0% (95% CI 83.3–96.7) in patients ≥80 years (Table S2).

In multivariate analysis (Table S2), age, laterality, grade, and stage were significant prognostic factors of DSS. However, patients with right lesions were associated with worse 5‐year DSS (72.3%, 95% CI 65.8–78.8; HR 1.65, 95% CI 1.10–2.48; *p* = 0.015). Older age seemed to be a protective factor in DSS. DSS benefits were evident in patients aged 60–69 years (HR 0.32, 95% CI 0.16–0.62; *p* = 0.001), 70–79 years (HR 0.16, 95% CI 0.06–0.41; *p* < 0.001), and ≥80 years (HR 0.32, 95% CI 0.14–0.71; *p* = 0.005). Although Kaplan–Meier survival analysis showed that patients who received aggressive treatment tended to associate with worse survival outcomes (Figure 3), there was no statistically significant difference in surgery, chemotherapy, or radiotherapy in multivariate analysis.

### 3.4 Primary breast angiosarcoma

Further analysis of primary breast angiosarcoma was performed. For this, 264 patients were identified by SEER sequence number. Multivariate analysis (Table S3) implicated age 60–69 and ≥80 years, right lesion, and higher and more severe stage as OS risk factors. Black race was no longer a risk factor for primary breast angiosarcoma patients: 5‐year DSS 43.9% (95% CI 31.9–55.9; HR 1.24, 95% CI 1.10–2.48; *p* = 0.481). Multivariate analysis (Table S4) revealed worse 5‐year DSS for patients who received mastectomy: 49.8% (45.7%–53.9%; HR 4.02, 95% CI 1.11–14.61; *p* = 0.034). Patients ≥80 years of age with right lesions and higher and more severe stages were associated with worse survival. Compared with the mastectomy group, the Kaplan–Meier survival analysis in OS and DSS (Figure S6A,B) showed that patients who received conserving surgery associated with better OS (log‐rank *p* = 0.002) and DSS (log‐rank *p* < 0.001). Constituent ratio and chi‐squared test data (Table 2) revealed significantly higher values in the mastectomy group concerning the proportion of patients with higher tumor grade (grade III: mastectomy 21.6% vs. conserving surgery 13.3%, *p* = 0.006) and more severe stage (regional disease: mastectomy 19.1% vs. conserving surgery 6.7%, *p* = 0.025). In addition, in primary angiosarcoma, laterality remained a factor affecting the prognosis, prompting further analysis of the constituent ratios. Consistent with the previous results, the difference between the constituent ratios of the two groups was not statistically significant (Table S5).

**TABLE 2:** Constituent ratio and chi‐squared test of primary breast angiosarcoma patients treated by conserving surgery or mastectomy.

Characteristic	*N*	Conserving surgery (%)	Mastectomy (%)	*χ* ^2^	*p*
*N*	222	60	162		
Age of diagnosis (years)					
≤39	69	14 (23.3)	55 (34.0)	6.987	0.137
40–59	76	17 (28.3)	59 (36.4)		
60–69	30	12 (20.0)	18 (11.1)		
70–79	30	11 (18.3)	19 (11.7)		
≥80	17	6 (10.0)	11 (6.8)		
Race					
White	180	46 (76.7)	134 (82.7)	2.295	0.489
Black	15	4 (6.7)	11 (6.8)		
Other	25	9 (15.0)	16 (9.9)		
Unknown	2	1 (1.7)	1 (0.6)		
Diagnosis (years)					
1975–1999	72	19 (31.7)	53 (32.7)	0.022	0.989
2000–2009	84	23 (38.3)	61 (37.7)		
2010–2016	66	18 (30.0)	48 (29.6)		
Laterality					
Left	117	34 (56.7)	83 (51.2)	0.518	0.472
Right	105	26 (43.3)	79 (48.8)		
Grade					
I	37	11 (18.3)	26 (16.0)	14.346	0.006
II	59	18 (30.0)	41 (25.3)		
III	43	8 (13.3)	35 (21.6)		
IV	40	4 (6.7)	36 (22.2)		
Unknown	43	19 (31.7)	24 (14.8)		
Stage					
Localized	163	48 (80.0)	115 (71.0)	8.797	0.025
Regional	35	4 (6.7)	31 (19.1)		
Distant	15	3 (5.0)	12 (7.4)		
Unknown	9	5 (8.3)	4 (2.5)		
Chemotherapy					
No/unknown	155	52 (86.7)	103 (63.6)	11.075	0.001
Yes	67	8 (13.3)	59 (36.4)		
Radiotherapy					
No/unknown	152	45 (75.0)	107 (66.0)	1.625	0.202
Yes	70	15 (25.0)	55 (34.0)		

## 4 DISCUSSION

Published studies on breast angiosarcoma have been scant due to the rarity of the disease. However, in recent years, because of the increasing incidence and uncertainty of treatment methods, researchers have realized that more prospective analysis and various studies are needed to inform the best management decisions for breast angiosarcoma. In this study, we screened breast angiosarcoma data in the SEER database from 1975 to 2016 and analyzed the factors influencing the poor prognosis of this disease.

We selected patients with primary breast angiosarcoma as much as possible from the cohort. The lack of a clear definition of primary and secondary breast angiosarcoma in the SEER database could introduce a selection bias when grouping according to whether patients were diagnosed based on a previous tumor.^11^ To evaluate the survival outcomes more comprehensively, we calculated both OS and DSS. DSS was accurate in evaluating the survival of patients with primary disease because there was no need to consider the impact of breast cancer on the cause of death. However, analyzing the entire cohort would result in a lower DSS. This bias was also the major limitation of this study.

We observed opposite influences of age for OS and DSS. Older age was a risk factor in OS, but was protective in DSS. A systematic review of 222 patients diagnosed with radiation‐associated breast angiosarcoma after breast cancer (secondary breast angiosarcoma) from 74 articles revealed that age was a significant prognostic factor on OS (HR 1.03, 95% CI 1.00–1.07; *p* = 0.048).^12^ Also, another retrospective study of the SEER database reported that compared with patients <60 years of age, patients ≥60 years had a worse OS in both primary (HR 1.54, 95% CI 1.04–2.28; *p* = 0.032) and secondary breast angiosarcoma (HR 1.91, 95% CI 1.07–3.42; *p* = 0.03).^5^ The impact of age on OS evident in these prior studies is the same as found in the present study. However, we were more interested in the DSS results. Undeniably, the small number of patients and some confounding factors (such as the subtype) could have affected DSS results in our study. Patients diagnosed with secondary breast angiosarcoma represent a relatively older age group and most have a history of breast cancer. The pernicious impact of secondary breast angiosarcoma on breast cancer has been previously demonstrated.^13^ All these factors will influence the assessment of age on survival outcome. In our research, both the OS and DSS findings showed that the influence of older age on survival was negative. Therefore, combined with the result in the DSS analysis, the effect of age on the survival of patients with secondary breast angiosarcoma was not clear. Tumor‐related survival and other indicators should also be considered.

Surprisingly, we found that right lesion was a significant risk factor for patients with breast angiosarcoma in OS (HR 1.34, 95% CI 1.09–1.64; *p* = 0.005) and DSS (HR 1.65, 95% CI 1.10–2.48; *p* = 0.015). The same result was evident in the analysis of primary breast angiosarcoma. The *χ* ^2^ test was performed to further compare the characteristics and differences between the left and right groups. No statistically significant difference was found. Due to the rarity of the disease and the lack of some data from SEER database (such as tumor size, margin status, and other data), our comparisons of patient characteristics were limited. The influence of laterality on patients with breast angiosarcoma has hardly been studied. Therefore, we cannot state whether our discovery is universal or not based on the results of other studies. Nevertheless, we tried to evaluate the result compared to the effect of laterality on breast cancer. Compared with patients irradiated for right‐sided breast cancer, patients with left lesions have been reported to have worse survival because of higher cardiac radiation dose.^14^ Generally, laterality shows no significant predictive effect on survival. A multivariate analysis of 305,443 women suggested that laterality was not a predictor of breast cancer‐specific mortality (*p* = 0.331).^15^ Another single‐center prospective study showed patients with right‐sided tumors had a better OS compared to their left‐sided counterparts in the luminal A subtype (*p* = 0.0491).^16^ The authors also analyzed the angiogenic factors and found a significantly higher concentration of vascular endothelial growth factor‐A (VEGF‐A) (*p* = 0.0136) in patients with breast cancer localized in the left breast.^16^ Although we did not find corroborating evidence, we noted that the concentration of angiogenesis‐regulated factors localized in the breast may be different between patients with left‐ and right‐side breast cancer. VEGF‐ and VEGF receptor (VEGFR)‐related genomic alterations are common in angiosarcoma. VEGFR‐3 amplification, VEGFR‐2 mutations, and VEGF/VEGFR family overexpression have all been reported to have different frequency alterations.^17^ Lower VEGFR‐2 expression can reportedly lead to poorer OS (HR 5.16, 95% CI 1.40–19.04; *p* = 0.014).^18^ Consequently, the difference between VEGF and VEGFR expression in left‐ and right‐side breast angiosarcoma deserves to be studied. In addition, given that hematogenous metastasis is the main means of spread of breast angiosarcoma,^19^, ^20^ we propose the possibility that right‐sided breast angiosarcoma has a greater tendency of metastasis because of the unique vascular anatomy characteristic of the right side of the body. Moreover, based on the etiology of Stewart–Treves syndrome, we speculate that the regional lymphedema and lymphatic reflux status of right‐handed patients may also have an impact on survival. When medical staff face patients with breast angiosarcoma, lymphography seems to be able to further reveal the state of left and right breast and body lymphatic vessels, which may predict the prognosis of patients. Takumi et al. successfully obtained clear images of the superficial lymph circulation of the breast using Indocyanine Green lymphography. According to their previous Indocyanine Green lymphography staging of breast lymphedema, higher staging could be related to higher incidence of symptoms related to breast lymphedema.^21^ Nevertheless, a review by Sato and Yamamoto described that postoperative lymphedema is not a significant prognostic factor for patients with secondary breast angiosarcoma.^22^ The various possibilities need to be verified and their underlying mechanisms need to be clarified by further studies.

In some early studies, the predictive effect of grade on prognosis was not so clear. A retrospective analysis of 49 cases with primary breast angiosarcoma did not reveal statistically significant differences between grade and the rates of local recurrence or distant metastases (*p* > 0.05).^23^ With the continuous improvement of the classification system and increased study scale, more recent published data have shown that histologic grade and stage are significant prognostic factors. A retrospective study involving the SEER database on primary breast angiosarcoma found a statistically significant difference (*p* < 0.001) in median OS between localized grade I and grade II (both not reached) versus grade III (36 months).^24^ Another study of 36 cases of primary breast angiosarcoma in China also demonstrated the prognostic effect of tumor differentiation on disease‐free survival (*p* = 0.015).^25^ In a study of radiation‐associated angiosarcoma of the breast, Mito et al. concluded that high grade led to worse OS (*p* < 0.001).^26^ Our results are also consistent with the current largest sample size meta‐analysis including 975 breast angiosarcoma patients (380 primary, 595 secondary), in which patients with high‐grade tumor had worse recurrence‐free survival (HR 1.68, 95% CI 1.02–2.76).

There is no established optimal treatment program for patients with breast angiosarcoma. Especially for patients with primary breast angiosarcoma, clinicians usually deliver different degrees of radiotherapy or chemotherapy on the basis of surgical intervention with negative margins according to their own experience. In our study, surgery, chemotherapy, or radiotherapy were not significant prognostic factors in multivariate analysis. The trend in the Kaplan–Meier survival curve of DSS was even less optimistic. Patients who received more aggressive treatment (including both surgery and systemic adjuvant therapy) displayed a trend to worse DSS (Figure 3). In our analysis of primary breast angiosarcoma, mastectomy was even a prognostic risk factor (HR 4.02, 95% CI 1.11–14.61; *p* = 0.034). This was not convincing data, because only 15 primary breast angiosarcoma patients did not receive any surgery. This reflected the common use of mastectomy in patients with breast angiosarcoma. However, consistent with our results, no benefit of mastectomy on survival compared with breast‐conserving surgery was found in three studies.^5^, ^8^, ^27^ Another SEER study of breast sarcoma (angiosarcoma mostly) described that nonmetastatic breast sarcoma patients undergoing breast‐conserving surgery had a better OS (HR 1.96, 95% CI 1.48–2.61).^28^ The authors also emphasized that the type of surgery was not as important as a negative margin. Similar to these results, the Kaplan–Meier survival analysis in our study showed that patients who received conserving surgery were associated with better survival (Figure S6). However, subsequent analysis showed that the difference in the constituent ratio between the conserving surgery group and the mastectomy group was statistically significant. The proportion of patients with higher tumor grades and more severe stages was larger in the mastectomy group. Tumor grade and stage are important prognostic factors. Most importantly, due to the lack of data, we could not definitively evaluate the impact of tumor size on prognosis. The size of a breast tumor is definitely one of the factors that affect the selection of surgical methods. As a consequence, our results did not indicate a better prognosis for patients who underwent breast‐conserving surgery. Based on our analysis, we could only draw a conclusion that surgeons in this cohort study were more likely to choose mastectomy for patients with more advanced primary breast angiosarcoma. Nevertheless, it is difficult for clinicians to achieve R0 resection by breast‐conserving surgery in patients with breast angiosarcoma. Therefore, the selection of the best surgery remains controversial. When it comes to the choice of surgical methods, tumor size, histological grade, and stage of primary breast angiosarcoma should be considered by surgeons. More research on surgical treatment of primary breast angiosarcoma is needed.

The effects of systemic adjuvant treatment (including chemotherapy and radiotherapy) for patients with breast angiosarcoma are being studied. In the analysis of 58 patients with nonmetastatic breast angiosarcoma; chemotherapy led to a significantly better OS in secondary breast angiosarcoma group (*p* = 0.043).^29^ A retrospective study of cutaneous angiosarcoma showed that compared with patients who received taxane plus radiotherapy, patients who received taxane plus radiotherapy along with maintenance chemotherapy had an improved OS.^30^ Recently, treatments targeting programmed cell death protein 1 by the pembrolizumab monoclonal antibody,^31^ antibody to vascular endothelial growth factor,^32^ and other immune preparations have become the focus of studies. The effects of these drugs on angiosarcoma are being evaluated in clinical trials. More studies of the efficacy of chemotherapy and targeted drugs are needed.

Due to the paucity of studies on breast angiosarcoma, the effect of radiotherapy remains unknown. A few reports have indicated the increased efficacy of radiotherapy.^12^ In a case report on a patient with primary breast angiosarcoma, although the tumor size was large and the margin was positive, the patient survived with a 66‐month disease‐free period after surgery plus radiotherapy.^33^ Despite the limited value of the data as a reference due to the study scale, the multidisciplinary management of the patient is notable. Feinberg et al. compared the survival of patients with breast angiosarcoma (radiation‐associated) treated by sarcoma service and local hospitals. The findings indicate that specialist management of the disease may lead to a better DSS.^34^ Establishment of a multidisciplinary team of specialists on sarcoma can also be a good way to improve the diagnosis and treatment of breast angiosarcoma.

## 5 CONCLUSION

Angiosarcoma of the breast had a poor prognosis. In our study, age, laterality, histologic grade, and stage were significant prognostic factors. Why patients with angiosarcoma of the right breast had a worse prognosis remains equivocal. Clarification of the relationship between laterality and prognosis of patients with breast angiosarcoma is needed. Unfortunately, especially for primary breast angiosarcoma, concerning curative treatment, receiving more aggressive treatment is not a protective survival factor. In the analysis of DSS, the survival outcome of patients who received active treatment showed an even worse trend. Mastectomy was adopted more often by surgeons in this cohort study for patients with advanced primary breast angiosarcoma. For patients with breast angiosarcoma, the prospect of systemic adjuvant therapy deserves further investigation.

## AUTHOR CONTRIBUTIONS

**Junfeng Li:** Conceptualization (equal); formal analysis (equal); investigation (equal); methodology (equal); writing – original draft (lead); writing – review and editing (equal). **Yunhai Li:** Conceptualization (equal); formal analysis (equal); investigation (equal); methodology (equal); writing – original draft (supporting); writing – review and editing (equal). **Yuanyuan Wang:** Software (equal). **Zhao Li:** Software (equal). **Huan Zhang:** Resources (equal). **Yidan Gao:** Resources (equal). **Jinxiang Tan:** Conceptualization (equal); writing – review and editing (equal).

## FUNDING INFORMATION

This study was supported in part by the National Natural Science Foundation of China (No. 81102008) and the Chongqing Human Resources and Social Security Bureau returned overseas students selected funding project (2013012). The funding source had no role in the design and conduct of the study; collection, management, analysis, and interpretation of the data; preparation, review, or approval of the manuscript; and decision to submit the manuscript for publication.

## CONFLICT OF INTEREST STATEMENT

The authors declare that they have no competing interests.

## CONSENT FOR PUBLICATION

Not applicable.

## Supporting information


Table S1.

## Data Availability

LJF and LYH had full access to all of the data in the study and take responsibility for the integrity of the data and the accuracy of the data analysis.
