# Supplementary material for: Clinicopathological characteristics and survival outcomes in patients with angiosarcoma of breast
Source: Cancer Med. 2023 May 4;12(12):13397–407. doi: 10.1002/cam4.6042 (PMC10315782; doi:10.1002/cam4.6042)
Supplement: Supplementary file 1 — Table S1. [file CAM4-12-13397-s001.docx]

**Supplements**

**eTable 1.** Characteristics of Patients with Breast Angiosarcoma from the Surveillance, Epidemiology, and End Results Database, 1975-2016.

**eTable 2.** Five-year DSS and hazard ratios (HRs) for DSS in breast angiosarcoma.

**eTable 3.** Five-year OS and hazard ratios (HRs) for OS in primary breast angiosarcoma.

**eTable 4.** Five-year DSS and hazard ratios (HRs) for DSS in primary breast angiosarcoma.

**eTable 5.** The constituent ratio and chi-square test of primary breast angiosarcoma patients with different laterality.

**eFigure 1.** Flowchart for patients identified from Surveillance, Epidemiology, and End Results database.

**eFigure 2.** Kaplan-Meier survival curves in all patients.

**eFigure 3.** Kaplan-Meier survival curves for treatment in OS.

**eFigure 4.** Kaplan-Meier survival curves for clinic features in DSS.

**eFigure 5.** Kaplan-Meier survival curves for oncology characteristics in DSS.

**eFigure 6.** Kaplan-Meier overall survival (OS) and disease-specific survival (DSS) curves for different surgical methods in primary breast angiosarcoma patients.

**eTable 1. Characteristics of Patients with Breast Angiosarcoma from the Surveillance, Epidemiology, and End Results Database, 1975-2016.**

| Characteristic | All patients | |
| --- | --- | --- |
|  | n | % |
| Total N | 656 | 100 |
| Age |  |  |
| ≤39 | 90 | 13.72 |
| 40-59 | 140 | 21.34 |
| 60-69 | 128 | 19.51 |
| 70-79 | 142 | 21.65 |
| ≥80 | 156 | 23.78 |
| Sex |  |  |
| Male | 3 | 0.46 |
| Female | 653 | 99.54 |
| Race |  |  |
| White | 567 | 86.43 |
| Black | 41 | 6.25 |
| Other | 44 | 6.71 |
| Unknown | 4 | 0.61 |
| Diagnosis (years) |  |  |
| 1975-1999 | 153 | 23.32 |
| 2000-2009 | 279 | 42.53 |
| 2010-2016 | 224 | 34.15 |
| Laterality |  |  |
| Left | 337 | 51.37 |
| Right | 317 | 48.32 |
| Bilateral (single primary) | 2 | 0.30 |
| Grade |  |  |
| I | 73 | 11.13 |
| II | 104 | 15.85 |
| III | 150 | 22.87 |
| IV | 154 | 23.48 |
| Unknown | 175 | 26.68 |
| Stage |  |  |
| Localized | 345 | 52.59 |
| Regional | 207 | 31.55 |
| Distant | 35 | 5.34 |
| Unknown | 69 | 10.52 |
| Surgery |  |  |
| No | 45 | 6.86 |
| Conserving surgery | 91 | 13.87 |
| Mastectomy | 480 | 73.17 |
| Surgery, NOS | 35 | 5.34 |
| Unknown | 5 | 0.76 |
| Chemotherapy |  |  |
| No/Unknown | 494 | 75.30 |
| Yes | 162 | 24.70 |
| Radiotherapy |  |  |
| No/Unknown | 531 | 80.95 |
| Yes | 125 | 19.05 |
| Vital status |  |  |
| Alive | 257 | 39.18 |
| Dead of this cancer | 107 | 16.31 |
| Dead of other cause | 288 | 43.90 |
| Dead (missing/unknown COD) | 4 | 0.61 |
| Abbreviations: NOS, not otherwise specified; COD, cause of death. | | |

**eTable 2. Five-year DSS and hazard ratios (HRs) for DSS in breast angiosarcoma.**

| Variable | 5-year DSS (95% CI) | Univariate analysis | | | Multivariate analysis | | |
| --- | --- | --- | --- | --- | --- | --- | --- |
|  |  | HR | 95% CI | *P*-value | HR | 95% CI | *P*-value |
| Age of diagnosis (years) |  |  |  |  |  |  |  |
| ≤39 | 57.2% (45.8%-68.6%) | 1.00 | Reference |  | 1.00 | Reference |  |
| 40-59 | 73.2% (65.0%-81.4%) | 0.58 | 0.37-0.92 | 0.02 | 0.61 | 0.37-1.01 | 0.053 |
| 60-69 | 79.9% (70.9%-88.9%) | 0.35 | 0.20-0.62 | <0.001 | 0.32 | 0.16-0.62 | 0.001 |
| 70-79 | 94.8% (90.7%-98.9%) | 0.12 | 0.05-0.29 | <0.001 | 0.16 | 0.06-0.41 | <0.001 |
| ≥80 | 90.0% (83.3%-96.7%) | 0.28 | 0.14-0.54 | <0.001 | 0.32 | 0.14-0.71 | 0.005 |
| Race |  |  |  |  |  |  |  |
| White | 78.9% (74.6%-83.2%) | 1.00 | Reference |  | 1.00 | Reference |  |
| Black | 77.3% (60.4%-94.2%) | 1.13 | 0.53-2.45 | 0.75 | 1.22 | 0.51-2.91 | 0.65 |
| Other | 71.6% (55.5%-87.7%) | 1.22 | 0.64-2.35 | 0.55 | 1.25 | 0.63-2.48 | 0.53 |
| Diagnosis (years) |  |  |  |  |  |  |  |
| 1975-1999 | 70.6% (62.8%-78.4%) | 1.00 | Reference |  | 1.00 | Reference |  |
| 2000-2009 | 82.8% (77.3%-88.3%) | 0.52 | 0.34-0.80 | 0.003 | 0.75 | 0.45-1.25 | 0.27 |
| 2010-2016 | 80.1% (71.5%-88.7%) | 0.62 | 0.38-1.04 | 0.07 | 1.01 | 0.56-1.81 | 0.99 |
| Laterality |  |  |  |  |  |  |  |
| Left | 83.5% (78.6%-88.4%) | 1.00 | Reference |  | 1.00 | Reference |  |
| Right | 72.3% (65.8%-78.8%) | 1.57 | 1.07-2.30 | 0.02 | 1.65 | 1.10-2.48 | 0.015 |
| Grade |  |  |  |  |  |  |  |
| I | 82.0% (71.8%-92.2%) | 1.00 | Reference |  | 1.00 | Reference |  |
| II | 80.5% (71.9%-89.1%) | 1.38 | 0.67-2.85 | 0.38 | 1.13 | 0.54-2.37 | 0.74 |
| III | 67.9% (57.5%-78.3%) | 2.27 | 1.14-4.54 | 0.02 | 2.30 | 1.09-4.84 | 0.029 |
| IV | 80.3% (71.3%-89.3%) | 1.15 | 0.54-2.43 | 0.72 | 0.93 | 0.41-2.10 | 0.86 |
| Unknown | 81.4% (74.1%-88.7%) | 1.21 | 0.60-2.46 | 0.59 | 1.06 | 0.49-2.31 | 0.88 |
| Stage |  |  |  |  |  |  |  |
| Localized | 80.9% (76.2%-85.6%) | 1.00 | Reference |  | 1.00 | Reference |  |
| Regional | 80.1% (72.3%-87.9%) | 0.84 | 0.52-1.37 | 0.48 | 1.06 | 0.63-1.80 | 0.82 |
| Distant | 36.2% (14.8%-57.6%) | 7.10 | 4.03-12.51 | <0.001 | 6.19 | 3.14-12.24 | <0.001 |
| Unknown | 85.3% ( - ) | 0.65 | 0.24-1.79 | 0.41 | 1.26 | 0.45-3.54 | 0.66 |
| Surgery |  |  |  |  |  |  |  |
| No | 91.6% ( - ) | 1.00 | Reference |  | 1.00 | Reference |  |
| Conserving surgery | 87.3% (78.9%-95.7%) | 1.48 | 0.33-6.69 | 0.61 | 1.49 | 0.30-7.33 | 0.63 |
| Mastectomy | 77.4% (72.7%-82.1%) | 2.43 | 0.60-9.90 | 0.04 | 2.26 | 0.50-10.12 | 0.29 |
| Surgery, NOS | 64.7% (47.1%-82.3%) | 4.22 | 0.95-18.70 | 0.06 | 2.49 | 0.49-12.66 | 0.27 |
| Chemotherapy |  |  |  |  |  |  |  |
| No/Unknown | 82.2% (77.9%-86.5%) | 1.00 | Reference |  | 1.00 | Reference |  |
| Yes | 66.4% (57.0%-75.8%) | 2.08 | 1.40-3.07 | <0.001 | 1.18 | 0.74-1.88 | 0.48 |
| Radiotherapy |  |  |  |  |  |  |  |
| No/Unknown | 82.4% (78.3%-86.5%) | 1.00 | Reference |  | 1.00 | Reference |  |
| Yes | 62.7% (52.3%-73.1%) | 2.36 | 1.59-3.51 | <0.001 | 1.30 | 0.84-2.03 | 0.24 |
| Abbreviations: DSS, disease-specific survival; NOS, not otherwise specified. | | | | | | | |

**eTable 3. Five-year OS and hazard ratios (HRs) for OS in primary breast angiosarcoma.**

| Variable | 5-year OS (95% CI) | Univariate analysis | | | Multivariate analysis | | |
| --- | --- | --- | --- | --- | --- | --- | --- |
|  |  | HR | 95% CI | *P*-value | HR | 95% CI | *P*-value |
| Age of diagnosis (years) |  |  |  |  |  |  |  |
| ≤39 | 50.6% (44.8%-56.4%) | 1.00 | Reference |  | 1.00 | Reference |  |
| 40-59 | 59.1% (55.3%-62.9%) | 0.93 | 0.62-1.40 | 0.718 | 0.97 | 0.62-1.52 | 0.885 |
| 60-69 | 49.3% (41.1%-57.5%) | 1.37 | 0.84-2.24 | 0.202 | 1.99 | 1.16-3.40 | 0.012 |
| 70-79 | 57.1% (48.4%-65.8%) | 1.38 | 0.81-2.32 | 0.234 | 1.72 | 0.96-3.07 | 0.066 |
| ≥80 | 18.2% (10.0%-26.4%) | 3.46 | 2.04-5.88 | <0.001 | 5.19 | 2.83-9.52 | <0.001 |
| Race |  |  |  |  |  |  |  |
| White | 50.8% (47.3%-54.3%) | 1.00 | Reference |  | 1.00 | Reference |  |
| Black | 43.9% (31.9%-55.9%) | 1.46 | 0.86-2.50 | 0.166 | 1.24 | 0.68-2.24 | 0.481 |
| Other | 53.0% (43.3%-62.7%) | 0.89 | 0.54-1.47 | 0.648 | 1.23 | 0.72-2.11 | 0.447 |
| Diagnosis (years) |  |  |  |  |  |  |  |
| 1975-1999 | 58.1% (53.2%-63.0%) | 1.00 | Reference |  | 1.00 | Reference |  |
| 2000-2009 | 51.4% (46.0%-56.8%) | 1.30 | 0.89-1.88 | 0.173 | 1.09 | 0.71-1.68 | 0.704 |
| 2010-2016 | 40.5% (34.2%-46.8%) | 1.54 | 1.01-2.33 | 0.044 | 1.33 | 0.83-2.13 | 0.230 |
| Laterality |  |  |  |  |  |  |  |
| Left | 58.5% (54.2%-62.8%) | 1.00 | Reference |  | 1.00 | Reference |  |
| Right | 45.2% (40.6%-49.8%) | 1.36 | 1.00-1.85 | 0.054 | 1.51 | 1.09-2.12 | 0.015 |
| Grade |  |  |  |  |  |  |  |
| I | 64.0% (56.3%-71.7%) | 1.00 | Reference |  | 1.00 | Reference |  |
| II | 68.5% (62.5%-74.5%) | 1.05 | 0.59-1.86 | 0.872 | 1.34 | 0.73-2.47 | 0.351 |
| III | 29.5% (22.6%-36.4%) | 3.13 | 1.78-5.49 | <0.001 | 3.40 | 1.83-6.30 | <0.001 |
| IV | 28.8% (21.4%-36.2%) | 2.67 | 1.50-4.77 | 0.001 | 2.33 | 1.17-4.61 | 0.016 |
| Unknown | 55.3% (49.4%-61.2%) | 1.50 | 0.88-2.56 | 0.139 | 1.50 | 0.83-2.72 | 0.180 |
| Stage |  |  |  |  |  |  |  |
| Localized | 62.2% (58.5%-65.9%) | 1.00 | Reference |  | 1.00 | Reference |  |
| Regional | 19.5% (13.3%-25.7%) | 2.87 | 1.94-4.25 | <0.001 | 2.28 | 1.49-3.48 | <0.001 |
| Distant | 10.0% (3.3%-16.7%) | 7.46 | 4.50-12.35 | <0.001 | 10.26 | 5.51-19.09 | <0.001 |
| Unknown | 61.9% (49.6%-74.2%) | 1.14 | 0.59-2.19 | 0.695 | 1.61 | 0.76-3.40 | 0.210 |
| Surgery |  |  |  |  |  |  |  |
| No | 52.7% (38.6%-66.8%) | 1.00 | Reference |  | 1.00 | Reference |  |
| Conserving surgery | 72.1% (66.2%-78.0%) | 0.46 | 0.22-0.95 | 0.036 | 0.95 | 0.42-2.16 | 0.899 |
| Mastectomy | 43.8% (39.8%-47.8%) | 0.90 | 0.47-1.72 | 0.744 | 1.80 | 0.83-3.90 | 0.139 |
| Surgery, NOS | 50.3% (40.5%-60.1%) | 0.64 | 0.29-1.40 | 0.264 | 2.09 | 0.75-5.80 | 0.156 |
| Chemotherapy |  |  |  |  |  |  |  |
| No/Unknown | 56.2% (52.5%-59.9%) | 1.00 | Reference |  | 1.00 | Reference |  |
| Yes | 38.8% (33.0%-44.6%) | 1.38 | 0.98-1.94 | 0.063 | 1.01 | 0.66-1.54 | 0.961 |
| Radiotherapy |  |  |  |  |  |  |  |
| No/Unknown | 54.3% (50.5%-58.1%) | 1.00 | Reference |  | 1.00 | Reference |  |
| Yes | 44.9% (39.3%-50.5%) | 1.05 | 0.75-1.46 | 0.797 | 0.75 | 0.52-1.10 | 0.134 |
| Abbreviations: OS, overall survival; NOS, not otherwise specified. | | | | | | | |

**eTable 4. Five-year DSS and hazard ratios (HRs) for DSS in primary breast angiosarcoma.**

| Variable | 5-year DSS (95% CI) | Univariate analysis | | | Multivariate analysis | | |
| --- | --- | --- | --- | --- | --- | --- | --- |
|  |  | HR | 95% CI | *P*-value | HR | 95% CI | *P*-value |
| Age of diagnosis (years) |  |  |  |  |  |  |  |
| ≤39 | 53.4% (47.5%-59.3%) | 1.00 | Reference |  | 1.00 | Reference |  |
| 40-59 | 61.6% (56.3%-66.9%) | 0.85 | 0.54-1.32 | 0.460 | 0.80 | 0.49-1.32 | 0.381 |
| 60-69 | 56.3% (47.7%-64.9%) | 0.85 | 0.47-1.54 | 0.588 | 1.47 | 0.77-2.81 | 0.246 |
| 70-79 | 80.7% (73.6%-87.8%) | 0.40 | 0.17-0.94 | 0.036 | 0.52 | 0.21-1.29 | 0.159 |
| ≥80 | 43.0% (30.6%-55.4%) | 1.91 | 1.00-3.67 | 0.051 | 3.08 | 1.45-6.54 | 0.003 |
| Race |  |  |  |  |  |  |  |
| White | 57.1% (53.5%-60.7%) | 1.00 | Reference |  | 1.00 | Reference |  |
| Black | 68.7% (56.8%-80.6%) | 0.78 | 0.34-1.78 | 0.551 | 0.80 | 0.33-1.91 | 0.609 |
| Other | 60.5% (50.4%-70.6%) | 0.84 | 0.46-1.54 | 0.576 | 1.22 | 0.64-2.30 | 0.550 |
| Diagnosis (years) |  |  |  |  |  |  |  |
| 1975-1999 | 62.2% (57.2%-67.2%) | 1.00 | Reference |  | 1.00 | Reference |  |
| 2000-2009 | 60.9% (55.4%-66.4%) | 1.06 | 0.68-1.65 | 0.799 | 0.98 | 0.58-1.66 | 0.950 |
| 2010-2016 | 51.4% (44.7%-58.1%) | 1.32 | 0.82-2.12 | 0.251 | 1.38 | 0.80-2.39 | 0.246 |
| Laterality |  |  |  |  |  |  |  |
| Left | 67.2% (63.0%-71.4%) | 1.00 | Reference |  | 1.00 | Reference |  |
| Right | 49.5% (44.7%-54.3%) | 1.54 | 1.06-2.24 | 0.025 | 1.59 | 1.06-2.40 | 0.028 |
| Grade |  |  |  |  |  |  |  |
| I | 70.9% (63.5%-78.3%) | 1.00 | Reference |  | 1.00 | Reference |  |
| II | 74.0% (68.2%-79.8%) | 1.20 | 0.59-2.42 | 0.619 | 1.25 | 0.60-2.59 | 0.553 |
| III | 30.3% (23.3%-37.3%) | 3.93 | 2.01-7.68 | <0.001 | 3.57 | 1.72-7.40 | 0.001 |
| IV | 41.1% (31.8%-50.4%) | 2.45 | 1.20-5.03 | 0.014 | 1.36 | 0.59-3.10 | 0.470 |
| Unknown | 64.8% (58.8%-70.8%) | 1.40 | 0.71-2.78 | 0.334 | 1.15 | 0.53-2.48 | 0.720 |
| Stage |  |  |  |  |  |  |  |
| Localized | 67.7% (64.1%-71.3%) | 1.00 | Reference |  | 1.00 | Reference |  |
| Regional | 30.8% (22.8%-38.8%) | 2.66 | 1.66-4.26 | <0.001 | 2.13 | 1.27-3.56 | 0.004 |
| Distant | 13.4% (5.0%-22.2%) | 9.65 | 5.58-16.68 | <0.001 | 13.46 | 6.65-27.24 | <0.001 |
| Unknown | 77.1% (65.4%-88.8%) | 0.52 | 0.16-1.64 | 0.515 | 0.96 | 0.28-3.31 | 0.950 |
| Surgery |  |  |  |  |  |  |  |
| No | 82.5% (64.1%-71.3%) | 1.00 | Reference |  | 1.00 | Reference |  |
| Conserving surgery | 80.0% (74.6%-85.4%) | 0.83 | 0.24-2.90 | 0.776 | 1.69 | 0.43-6.66 | 0.457 |
| Mastectomy | 49.8% (45.7%-53.9%) | 2.31 | 0.73-7.33 | 0.154 | 4.02 | 1.11-14.61 | 0.034 |
| Surgery, NOS | 56.9% (46.6%-67.2%) | 1.78 | 0.50-6.32 | 0.371 | 5.32 | 1.14-24.75 | 0.0333 |
| Chemotherapy |  |  |  |  |  |  |  |
| No/Unknown | 65.7% (62.0%-69.4%) | 1.00 | Reference |  | 1.00 | Reference |  |
| Yes | 42.2% (36.2%-48.2%) | 1.98 | 1.35-2.91 | <0.001 | 1.07 | 0.66-1.73 | 0.781 |
| Radiotherapy |  |  |  |  |  |  |  |
| No/Unknown | 63.1% (59.3%-66.9%) | 1.00 | Reference |  | 1.00 | Reference |  |
| Yes | 50.2% (44.4%-56.0%) | 1.41 | 0.96-2.08 | 0.079 | 0.81 | 0.52-1.26 | 0.345 |
| Abbreviations: OS, overall survival; NOS, not otherwise specified. | | | | | | | |

**eTable 5. The constituent ratio and chi-square test of primary breast angiosarcoma patients with different laterality.**

| Characteristic | N | Left (%) | Right (%) | X^2^ | *P* |
| --- | --- | --- | --- | --- | --- |
| N | 264 | 139 | 125 |  |  |
| Age of diagnosis (years) |  |  |  |  |  |
| ≤39 | 80 | 42 (30.2) | 38 (30.4) | 4.477 | 0.345 |
| 40-59 | 91 | 41 (29.5) | 50 (40.0) |  |  |
| 60-69 | 38 | 24 (17.3) | 14 (11.2) |  |  |
| 70-79 | 33 | 19 (13.7) | 14 (11.2) |  |  |
| ≥80 | 22 | 13 (9.4) | 9 (7.2) |  |  |
| Race |  |  |  |  |  |
| White | 210 | 114 (82.0) | 96 (76.8) | 1.294 | 0.755 |
| Black | 19 | 9 (6.5) | 10 (8.0) |  |  |
| Other | 30 | 14 (10.1) | 16 (12.8) |  |  |
| Unknown | 5 | 2 (1.4) | 3 (2.4) |  |  |
| Diagnosis (years) |  |  |  |  |  |
| 1975-1999 | 101 | 49 (35.3) | 52 (41.6) | 1.934 | 0.380 |
| 2000-2009 | 89 | 52 (37.4) | 37 (29.6) |  |  |
| 2010-2016 | 74 | 38 (27.3) | 36 (28.8) |  |  |
| Grade |  |  |  |  |  |
| I | 42 | 25 (18.0) | 17 (13.6) | 5.335 | 0.255 |
| II | 62 | 31 (22.3) | 31 (24.8) |  |  |
| III | 45 | 21 (15.1) | 24 (19.2) |  |  |
| IV | 41 | 27 (19.4) | 14 (11.2) |  |  |
| Unknown | 74 | 35 (25.2) | 39 (31.2) |  |  |
| Stage |  |  |  |  |  |
| Localized | 186 | 103 (74.1) | 83 (66.4) | 2.083 | 0.555 |
| Regional | 41 | 18 (12.9) | 23 (18.4) |  |  |
| Distant | 20 | 10 (7.2) | 10 (8.0) |  |  |
| Unknown | 17 | 8 (5.8) | 9 (7.2) |  |  |
| Surgery |  |  |  |  |  |
| No | 15 | 9 (6.5) | 6 (4.8) | 1.063 | 0.786 |
| Conserving surgery | 60 | 34 (24.5) | 26 (20.8) |  |  |
| Mastectomy | 162 | 83 (59.7) | 79 (63.2) |  |  |
| Surgery, NOS | 27 | 13 (9.4) | 14 (11.2) |  |  |
| Chemotherapy |  |  |  |  |  |
| No/Unknown | 190 | 105 (75.5) | 85 (68.0) | 1.855 | 0.173 |
| Yes | 74 | 34 (24.5) | 40 (32.0) |  |  |
| Radiotherapy |  |  |  |  |  |
| No/Unknown | 182 | 97 (69.8) | 85 (68.0) | 0.098 | 0.754 |
| Yes | 82 | 42 (30.2) | 40 (32.0) |  |  |

**eFigure 1. Flowchart for patients identified from Surveillance, Epidemiology, and End Results database.**


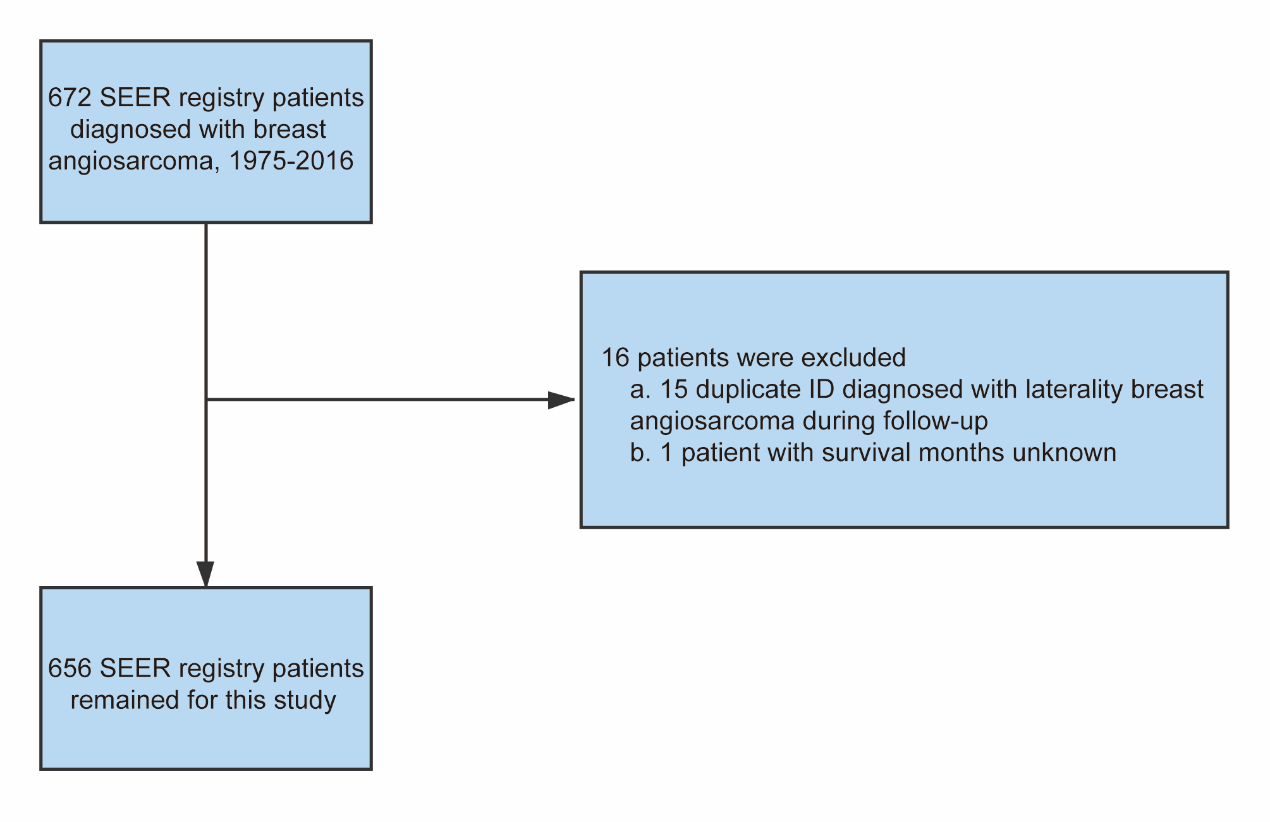


**eFigure 2.** **Kaplan-Meier survival curves in all patients.**


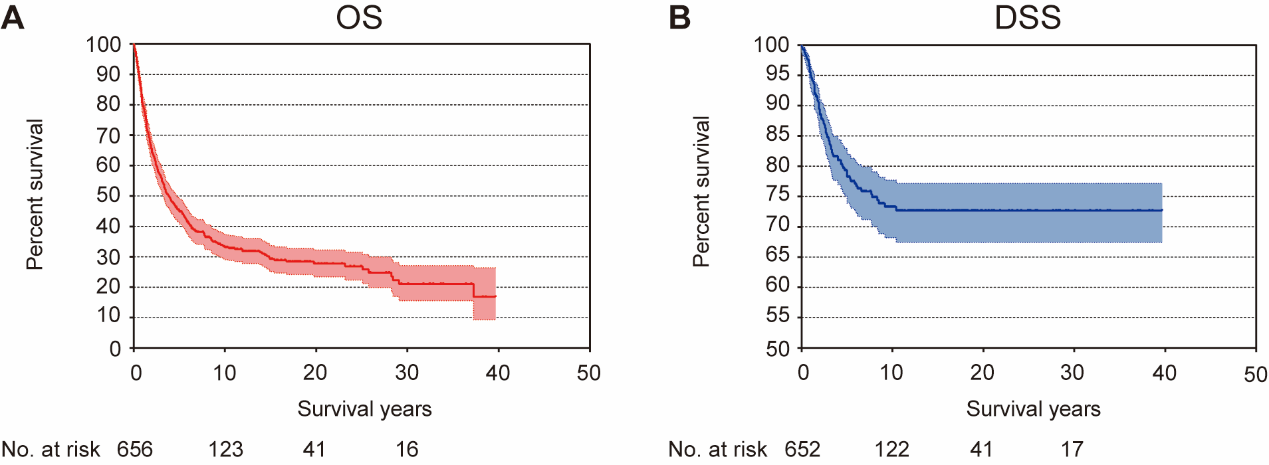


Stratified by (A) overall survival and (B) disease-specific survival.

**eFigure 3. Kaplan-Meier survival curves for treatment in OS.**


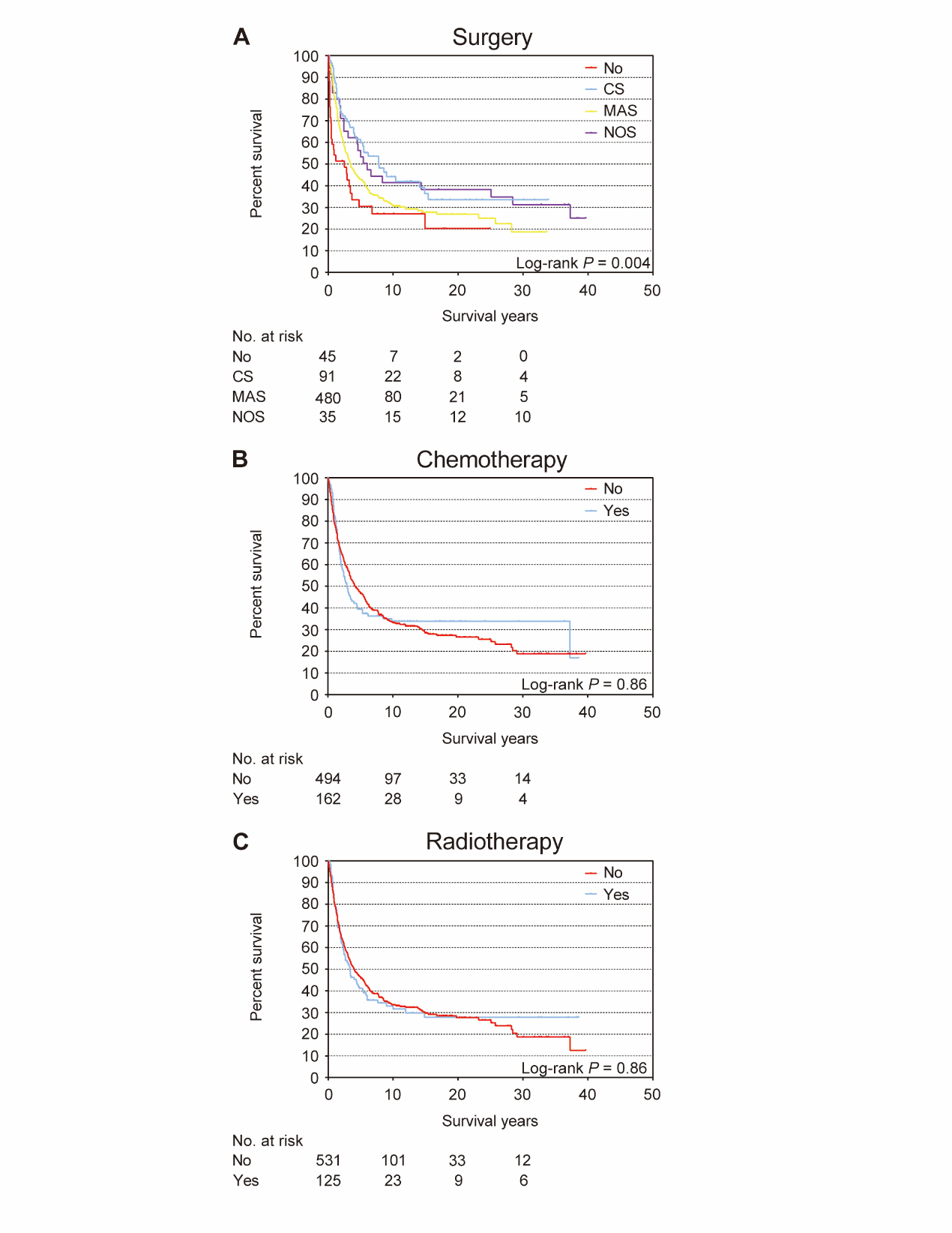


Stratified by (A) Surgery, (B) Chemotherapy and (C) Radiotherapy.

**eFigure 4.** **Kaplan-Meier survival curves for clinic features in DSS.**


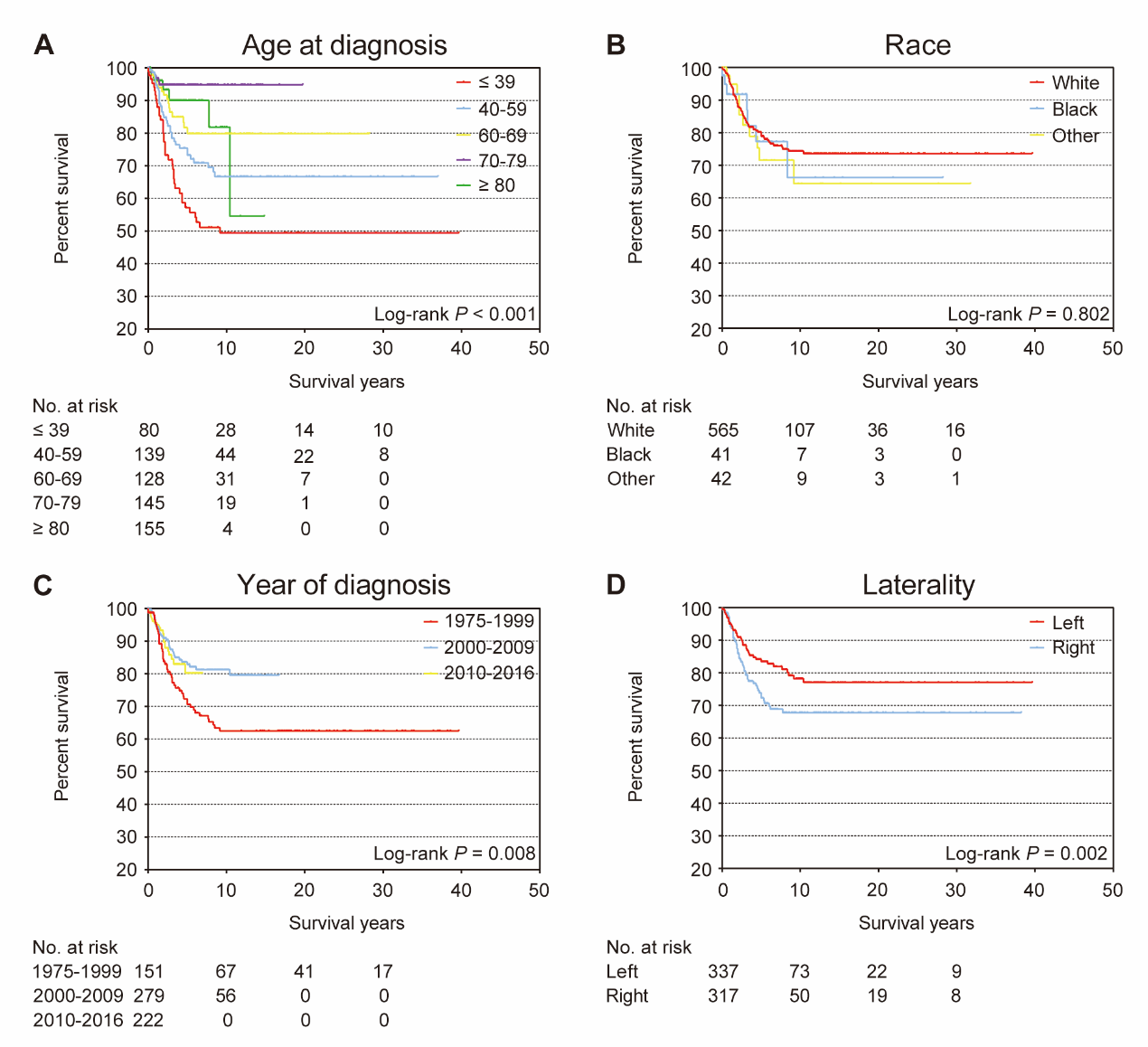


Stratified by (A) Age, (B) Race, (C) Year of diagnosis and (D) Laterality.

**eFigure 5.** **Kaplan-Meier survival curves for oncology characteristics in DSS.**


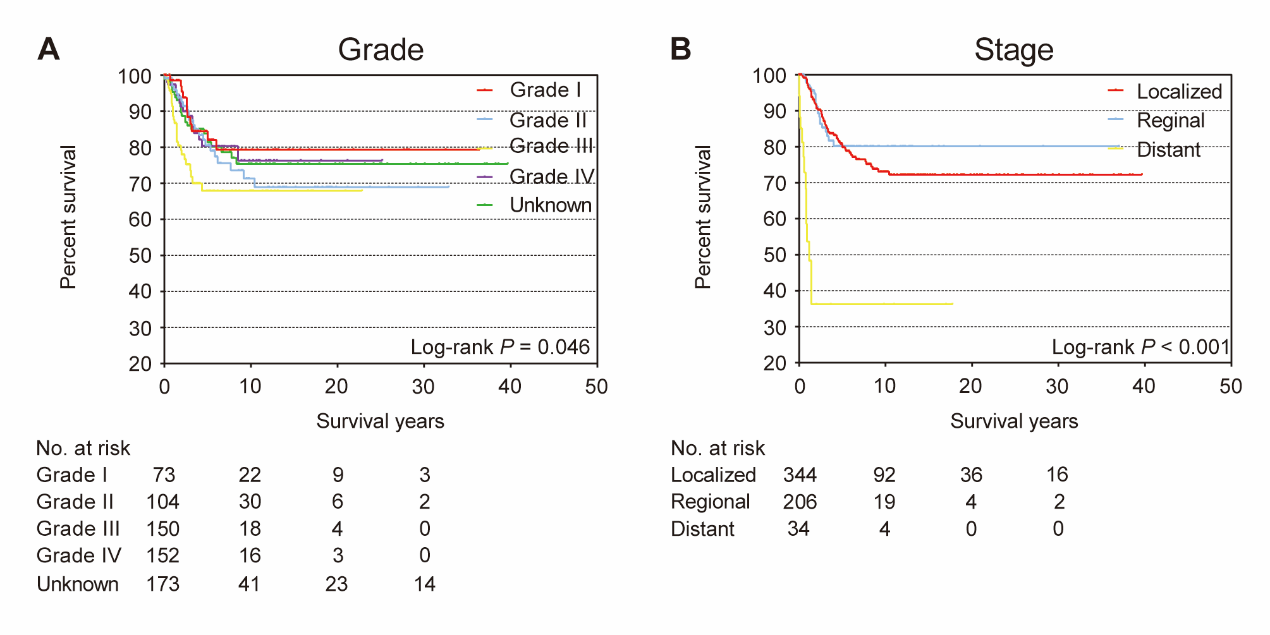


Stratified by (A) Grade and (B) Stage.

**eFigure 6.** **Kaplan-Meier overall survival (OS) and disease-specific survival (DSS) curves for different surgical methods in primary breast angiosarcoma patients.**


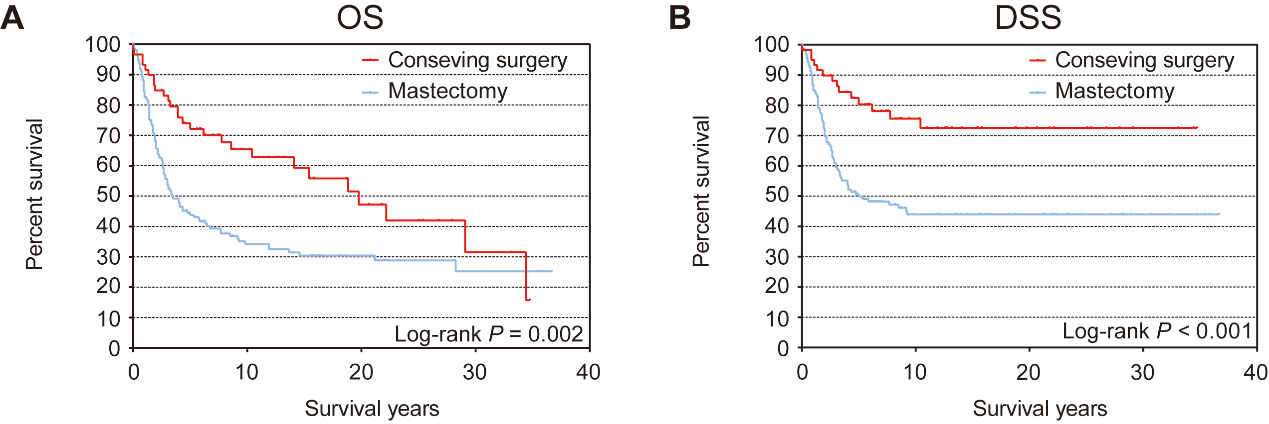


Stratified by (A) OS and (B) DSS.
